# Persistent Fear and Extended Swing Phase During Stair Descent Following Total Knee Arthroplasty: A Case Report

**DOI:** 10.7759/cureus.79608

**Published:** 2025-02-25

**Authors:** Maya Asaki, Masaya Anan

**Affiliations:** 1 Rehabilitation, Oita Oka Hospital, Oita, JPN; 2 Welfare and Health Science, Oita University, Oita, JPN

**Keywords:** anxiety, kinesiophobia, stair descent, surface electromyography, total knee arthroplasty

## Abstract

Total knee arthroplasty (TKA) is widely recognized as an effective procedure for reducing pain, improving activities of daily living (ADLs), and enhancing the quality of life in elderly patients with advanced knee osteoarthritis (KOA). However, many patients continue to have trouble with stair descent after TKA, a key functional movement that significantly affects postoperative satisfaction. This report describes the case of an 80-year-old woman with bilateral KOA who underwent left TKA. Despite undergoing postoperative rehabilitation, she continued to experience functional impairments and fear while stair descending, prompting the initiation of outpatient physiotherapy. Initial evaluations (78 days post-surgery) revealed limited range of motion (ROM) in the knee joint, muscle weakness, and high levels of anxiety and fear of movement, as measured by patient-reported outcome measures (PROMs). Surface electromyography (EMG) further identified abnormal muscle activity in the semitendinosus muscle and medial head of the gastrocnemius. Outpatient physiotherapy was performed twice a week and focused on improving ROM, strengthening the quadriceps and hamstrings, performing knee ROM exercises and mobilization, and providing guidance on independent home training. At the final evaluation (118 days post-surgery), knee ROM improved, and the PROMs score demonstrated enhanced self-efficacy. However, anxiety and fear of movement persisted. In terms of muscle strength, no significant improvement was observed. Additionally, the swing phase was extended, and EMG during stair descent revealed increased muscle activity in the semitendinosus and medial head of the gastrocnemius, suggesting compensatory protective muscle activity due to residual fear of movement. In addition to physical factors, such as knee ROM and quadriceps muscle strength, psychosocial factors, such as fear, anxiety, and depression, are critical components influencing outcomes after TKA. Residual fear is believed to delay ROM improvement and contribute to protective-like muscle activity. This case highlights the importance of a comprehensive rehabilitation approach addressing both physical and psychological aspects to optimize functional recovery in TKA patients with a pronounced fear of movement.

## Introduction

Total knee arthroplasty (TKA) is a widely performed procedure to reduce pain, enhance activities of daily living (ADLs), and improve the quality of life in elderly patients with advanced knee osteoarthritis (KOA) [1]. TKA provides excellent pain relief and functional improvement, with more than 90% of patients reporting satisfaction with the overall function of their artificial knee joint [2]. However, despite postoperative rehabilitation, approximately 60% of patients continue to experience difficulty descending stairs [3]. Stair descent requires the body to employ an eccentric contraction pattern to control acceleration due to the shift in the center of gravity. Compared to healthy individuals, patients with TKA exhibit insufficient eccentric contraction of the quadriceps femoris during stair descent [4]. As a result, stair descent imposes significant mechanical stress on the knee joint and requires controlled eccentric contraction of the quadriceps femoris, which contributes to the difficulty of this motion. This persistent functional challenge highlights the need for a deeper understanding of stair descent biomechanics and targeted rehabilitation strategies. The ratio of the stance phase to the swing phase during normal stair descent is 6:4, and numerous studies have focused on the biomechanics of the stance phase [5].

This case report focuses on a patient who had undergone left TKA for advanced bilateral KOA, a condition that caused significant pain and functional impairment. After being discharged home, the patient continued to face difficulty descending stairs despite outpatient physiotherapy. Key findings included an extended swing phase of the left lower limb during stair descent, accompanied by persistent anxiety and fear of movement. Anxiety and fear of movement can further contribute to declines in physical function, and these challenges underline the importance of addressing both physical and psychological aspects during post-TKA rehabilitation.

Here, we analyze the patient’s functional deficits using surface electromyography (EMG) during stair descent and assess these findings in the context of patient-reported outcome measures (PROMs). The patient reported increased muscle activity in the gastrocnemius during knee flexion and fatigue in the gastrocnemius during ADLs. To investigate the cause of this increased muscle activity, we conducted an evaluation using surface EMG. This case sheds light on the complex interplay between biomechanical and psychological factors in stair descent after TKA and discusses implications for optimizing rehabilitation interventions.

## Case presentation

An 80-year-old woman was independent in her ADLs before hospitalization and did not use any walking aids. However, she needed to hold onto the banister while ascending and stair descending, placing both feet on each step. Her height was 1.51 m, her mass was 53.5 kg, and her body mass index (BMI) was 23.6 kg/m^2^. She had a history of bilateral KOA, with a Kellgren-Lawrence classification of Grade III for the right knee and Grade IV for the left. Her chief complaint was pain in the knee joint during weight-bearing, and a left TKA was performed. The procedure was performed using the medial parapatellar approach, and a posterior-stabilized (PS) prosthesis was implanted.

**Figure 1:**
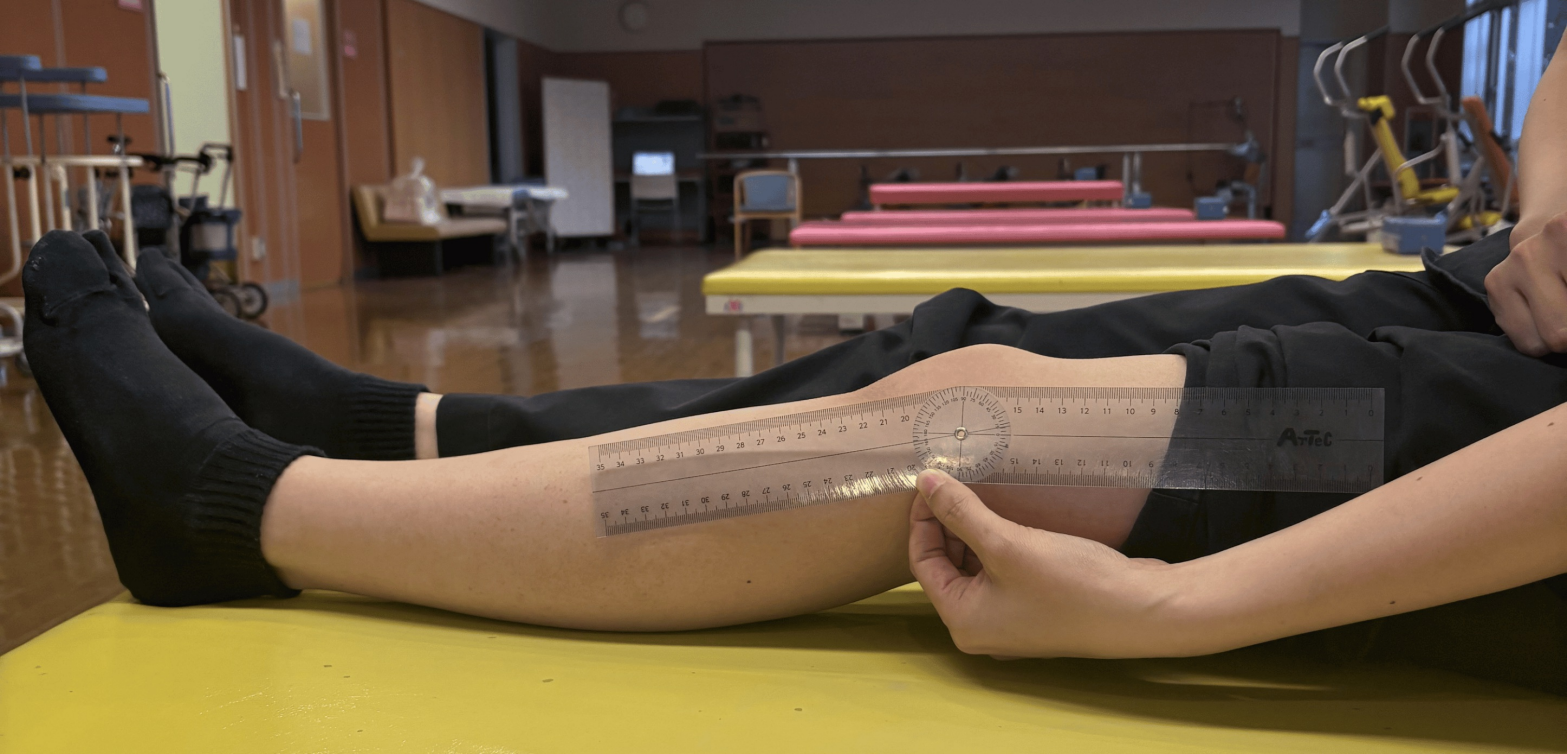
Photos of the goniometer used and the ROM evaluation ROM: range of motion

The evaluation results pre-surgery are shown in Table 1. The preoperative range of motion (ROM) measurements were as follows: 140° of flexion in the right knee, 125° of flexion in the left knee, -15° of extension in the right knee, and -20° of extension in the left knee. The goniometer and ROM evaluation photos used in Figure 1 are shown below. She scored 26 on the Pain Catastrophizing Scale score (PCS), 23 on the Hospital Anxiety Depression Scale score (HADS), and recorded Visual Analog Scale (VAS) scores of 70 mm at rest and 100 mm during walking. Her comfortable walking speed over 10 meters was 9.84 seconds.

**Table 1:** Evaluation results: pre-surgery, at discharge, 78 days post-surgery, and 114 days post-surgery ^-^The item was not measured or implemented. ROM: range of motion; VAS: visual analog scale; HHD: hand-held dynamometer; PROMs: patient-reported outcome measures; PCS: pain catastrophizing scale; HADS: hospital anxiety and depression Scale; TSK: Tampa Scale for Kinesiophobia; PSEQ: Pain Self-Efficacy Questionnaire

		Pre-surgery	At discharge	78 days post-surgery	114 days post-surgery
Functional evaluation (Left)	ROM (°)				
	Knee flexion	125	95	95	105
	Knee extension	-20	-15	-15	-15
	VAS (mm)				
	At rest	70	19	0	0
	During walking	100	32	30	0
	HHD (kgf/kg)				
	Knee flexion	-	-	0.11	0.14
	Knee extension	0.46	0.16	0.29	0.28
PROMs (points)	PCS	26	-	-	11
	HADS	23	-	-	35
	TSK	-	-	36	36
	PSEQ	-	-	28	54
Evaluation of stair descent	Duration of each step (s)	-	-	2.20	2.07
	Ratio of the stance phase of the left lower limb (%)	-	-	59.1	53.2
	Ratio of the swing phase of the left lower limb(%)	-	-	40.9	46.8

In our hospital's clinical rehabilitation program, basic movement exercises with a knee brace begin on the first postoperative day, while ROM and walking practice commence on the third postoperative day. Due to severe pain during movement and bearing weight, ROM and gait training for this patient also began on the third postoperative day. On postoperative day three, the ROM in the left knee was recorded as 60° of flexion and -15° of extension. During the postoperative period, she exhibited severe anxiety and emotional fluctuations, including persistent worry and mood swings, which contributed to a delay in the planned discharge date. Additionally, despite experiencing internal bleeding in her own leg, she became pessimistic upon observing that other TKA patients did not exhibit similar symptoms. She also developed emotional instability, including temporary insomnia and loss of appetite due to nighttime pain. In this case, the patient exhibited a strong fear of wound separation and increased pain, making it difficult to perform active knee ROM exercises. Therefore, we initiated automated assisted movement exercises in an anti-gravity position. Furthermore, based on the fear-avoidance model, ROM exercises were conducted within a pain-free range to prevent reinforcing pain-related fear and subsequent behavioral avoidance. In addition, postoperative cooling and compression therapy, which have been reported to be effective for pain management, were also implemented [6].

As she was able to perform the movements necessary for daily life, she was discharged from the hospital on her own 19 days after the surgery. At the time of discharge, the range of motion of her knee joint was 95° flexion, -15° extension, and her comfortable 10-meter walking speed improved to 9.20 seconds. She was instructed to continue knee flexion exercises in a sitting position, mobilization of the wound, stretching of the hamstrings and gastrocnemius muscles, and quadriceps setting exercises to improve knee extension. Additionally, she was advised on strategies to minimize knee joint strain and manage postoperative pain. However, by postoperative day 36, functional impairments and difficulty in stair descent persisted, and outpatient physiotherapy was initiated. Her main complaints during outpatient physiotherapy were fatigue localized to the gastrocnemius muscle after walking and significant difficulty with stair descent due to reduced control and confidence. The primary rehabilitation goal was to improve stair descent efficiency.

The purpose and content of this report were explained to the patient, and informed consent was obtained with assurances of privacy protection. Her progress was monitored through outpatient rehabilitation, and she provided consent for the rehabilitation evaluation and follow-up. This case report adheres to the CAse REport (CARE) guidelines and includes all necessary information.

Evaluation and data analysis

**Figure 2:**
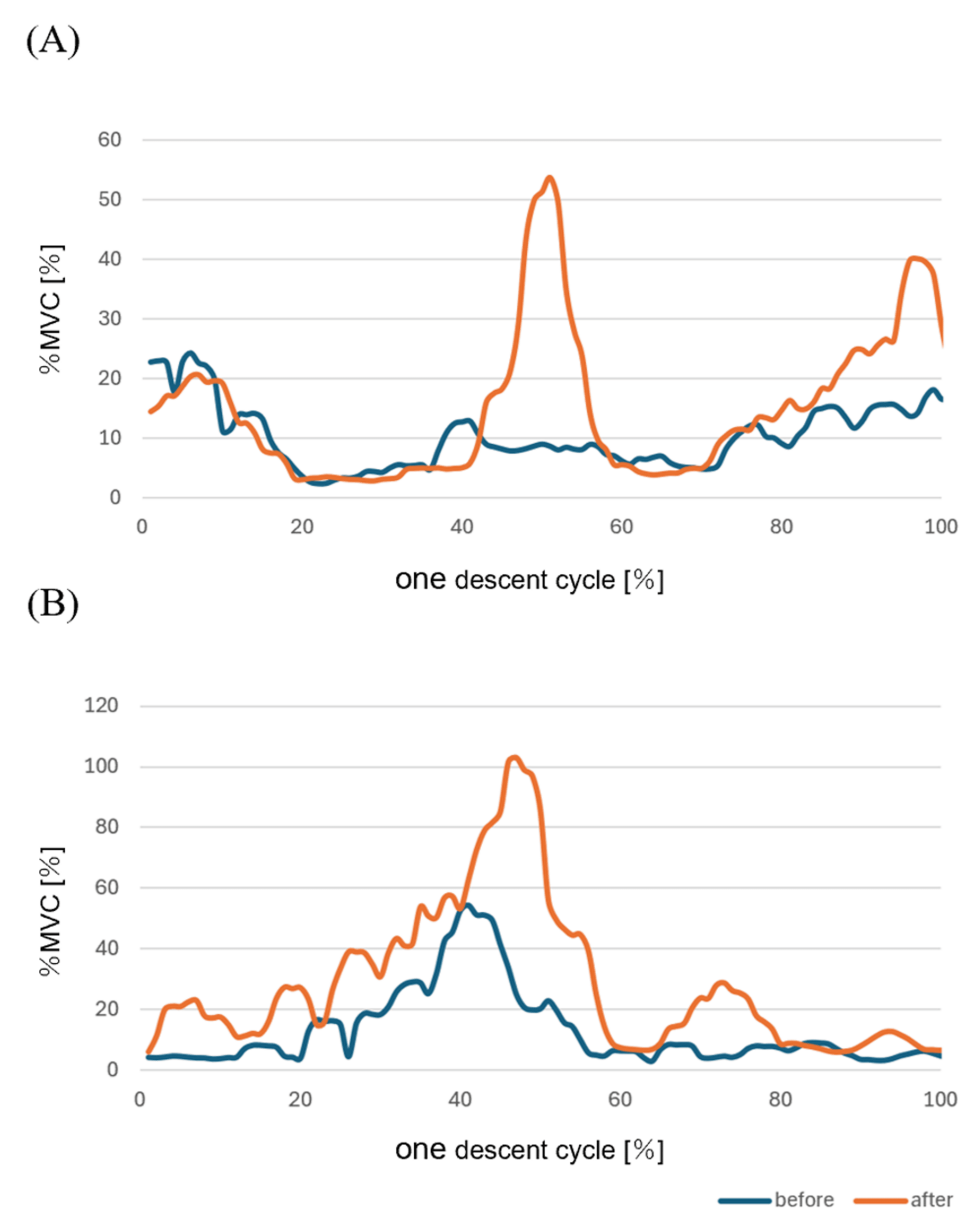
(A) Surface EMG of the semitendinosus muscle before and after invention. (B) Surface EMG of the medial head of the gastrocnemius muscle before and after invention MVC: maximum voluntary contraction; EMG: electromyography

At the initial evaluation (78 days after surgery), we assessed her ROM, muscle strength, pain, muscle activity during stair descent, and PROMs. The evaluation results and surface EMG are shown in Table 1 and Figure 2. The ROM was measured using a plastic goniometer (Artec, Japan), muscle strength was measured using a hand-held dynamometer (HHD) (μ-tas, Anima, Japan), and muscle activity was evaluated with surface EMG (TS-MYO, Trunk Solution, Japan). Muscle strength was calculated using the weight-bearing index (WBI). Stair descent was assessed using a 20 cm-high staircase from the patient’s daily environment and performed one step at a time while holding the right handrail. Pain was evaluated using VAS and was 0 mm both at rest and while walking.

**Figure 3:**
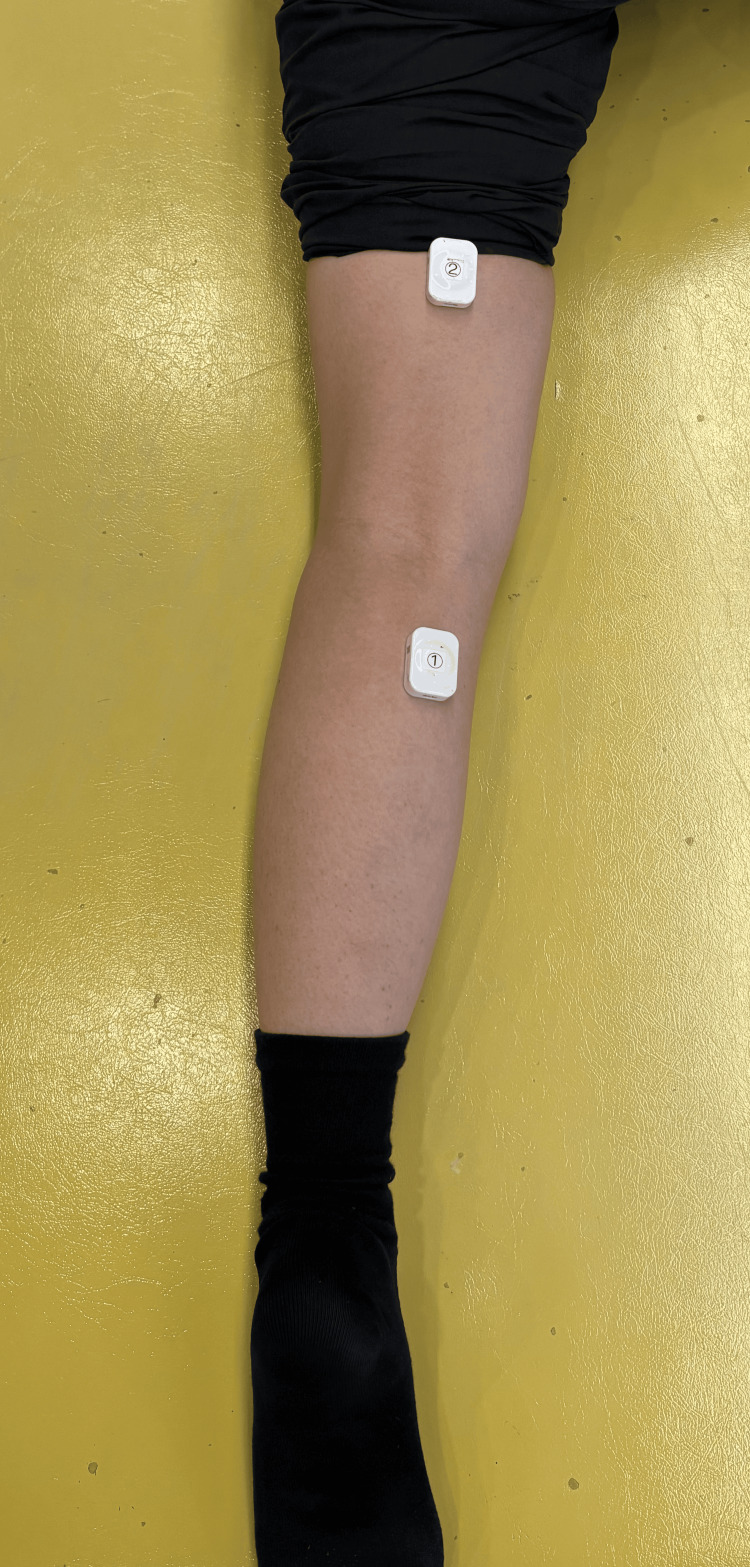
Surface EMG electrode placement sites for the left medial head of the gastrocnemius muscle and the left semitendinosus muscle EMG: electromyography

During automatic knee flexion, palpation and surface EMG confirmed greater activity in the medial head of the gastrocnemius muscle compared to the semitendinosus muscle. She also reported calf fatigue after walking. Consequently, surface EMG electrodes were attached to the left semitendinosus muscle and medial head of the gastrocnemius muscle. Figure 3 shows the surface EMG electrode placement sites, which were determined following the SENIAM guidelines [7]. The sampling frequency was set to 1 kHz, with a 20-450 Hz band-pass filter. Data were smoothed using a 100-ms root mean square (RMS) process, and each EMG waveform was normalized to the maximum voluntary isometric contraction (MVIC) over a five-second period. In addition, the data obtained using the surface electromyograph was normalized by time and then filtered at 20 Hz. EMG data during the stair descent were extracted for one descent cycle starting from the left toe-off after four steps. PROMs were evaluated using the Tampa Scale for Kinesiophobia (TSK) and Pain Self-Efficacy Questionnaire (PSEQ). The total score ranges for these assessments are 17-68 points for TSK and 0-60 points for PSEQ [8,9].

The ROM assessment revealed knee flexion of 95° and extension of -15°. During terminal flexion, she experienced a stretching sensation around the wound, while terminal extension caused stretching in the medial head of the gastrocnemius muscle. Muscle strength testing showed 0.11 kgf/kg for flexion and 0.29 kgf/kg for extension. EMG analysis indicated reduced semitendinosus muscle activity during the descent phase, particularly before initial left foot contact. Compared to the semitendinosus muscle, the medial head of the gastrocnemius exhibited increased muscle activity, suggesting that the patient had not yet developed an awareness of semitendinosus muscle contraction. The duration of each step during stair descent was 2.20 s, with a stance phase ratio of 59.1% and a swing phase ratio of 40.9%. At the end of the stance phase, she reported a stretching sensation at the front of the knee joint. PROMs evaluation revealed a TSK score of 36, indicating moderate kinesiophobia, and a PSEQ score of 28, suggesting reduced self-efficacy in managing pain. These scores aligned with the patient's statements: “I descend by placing both feet on each step because it seems like it might hurt” and “I stop immediately if it hurts.”

The peak knee joint flexion angle during the descent phase has been reported as 110.5°±13.0° [10]. In this case, she had not yet achieved the knee flexion ROM necessary for stair descent due to reduced rectus femoris extensibility and limited downward movement of the patella. Additionally, quadriceps muscle strength commonly decreases significantly in the months following TKA compared to preoperative values [11]. She had a decrease in isometric knee extension strength, and it was becoming difficult for her to descend stairs. In summary, her main impairments were reduced rectus femoris extensibility, leading to restricted knee flexion ROM due to limited patellar mobility, and decreased quadriceps muscle strength, likely caused by arthrogenic muscle inhibition resulting from postoperative inflammatory symptoms.

Physical therapy intervention

**Figure 4:**
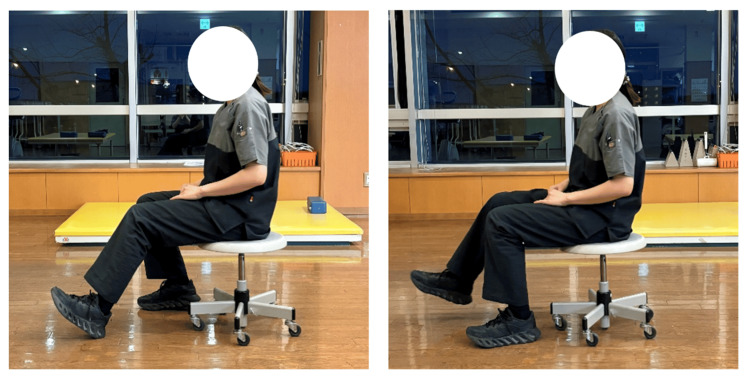
Pictures of chair walk

Outpatient physiotherapy was carried out twice a week for 40 minutes per session and focused on knee joint ROM training, quadriceps and hamstring muscle strengthening training, one-step descent movement practice, and standing balance practice. Each session was divided into two parts: the first 20 minutes were dedicated to ROM and muscle strengthening exercises, while the remaining 20 minutes focused on descent movement practice, balance training, and gait training. ROM exercises included mobilization of the skin and patella around the wound, as well as stretching of the tensor fasciae latae, rectus femoris, hamstring, and gastrocnemius muscles. The quadriceps muscle strengthening exercises included 10 repetitions of two sets of seated exercises that emphasized afferent and efferent nerve transmission, 20 repetitions of 2 sets of quadriceps muscle set exercises, and 10 repetitions of three sets of step-down exercises. For hamstrings strengthening, chair walks were performed. Figure 4 shows the chair walk.

During stair descent practice, the load was progressively adjusted in 5 cm increments based on step height. Additionally, to address movement-related fear associated with excessive knee flexion, step height, and foot positioning were modified to align with the patient's knee flexion ROM. Gait training was segmented according to the walking cycle: from initial contact to mid-stance, from terminal stance to pre-swing, and from pre-swing to initial contact. From a psychological perspective, and in accordance with the fear-avoidance model, ROM, and stair descent exercises were conducted within a pain-free range to prevent reinforcing pain-related fear and behavioral avoidance.

Additionally, she was instructed in independent training, which included skin and patella mobilization around the surgical area, stretching of the rectus femoris, gastrocnemius, and tensor fasciae latae muscles, and quadriceps strengthening exercises. She was able to follow the exercise regimen without experiencing increased pain. To prevent pain exacerbation due to a sudden increase in activity, pacing strategies were introduced to regulate walking duration and exercise volume.

Results of the intervention

The evaluation results of the final evaluation (114 days post-surgery) and surface EMG are shown in Table 1 and Figure 2. At the final evaluation, the knee ROM improved to 105° of flexion, but there was no change in -15° of extension. Muscle strength increased to 0.14 kgf/kg for knee flexion and decreased to 0.28 kgf/kg for extension. Before the left initial foot contact during stair descent, muscle activity in the semitendinosus muscle and medial head of the gastrocnemius increased. Additionally, the patient reported a reduced stretching sensation around the wound area and noted that stair descent felt easier due to the increased knee flexion angle in the late phase of left stance during descent. The step cycle time during stair descent was 2.07 s, with the stance phase ratio reduced to 53.2% and the swing phase ratio increased to 46.8%. Although the overall descent phase duration was reduced, the increased swing phase ratio suggested a decline in movement quality. This was likely due to the emergence of protective contractions in the semitendinosus muscle and medial head of the gastrocnemius during the swing phase.

In the PROMs evaluation, the TSK score remained at 36 points, while the PSEQ score improved significantly to 54 points. The PCS score decreased to 11 points, indicating reduced pain catastrophizing, and the HADS score was recorded as 35 points.

## Discussion

In this case, we analyzed the patient’s functional impairment using EMG to evaluate stair descent mechanics and assessed it in relation to PROMs. The results showed a prolonged left swing phase during stair descent, along with increased muscle activity in the semitendinosus and medial head of the gastrocnemius. In terms of PROMs, we observed improvements in pain catastrophizing and self-efficacy for pain management; however, anxiety, depression, and movement-related fear persisted. Regarding physical function, knee flexion ROM improved, but muscle strength did not change.

The increased duration of the swing phase in stair descent may have been influenced by elevated muscle activity in the semitendinosus and medial head of the gastrocnemius. Previous studies have reported that semitendinosus activity during the swing phase contributes to decelerating the extended leg, while gastrocnemius activity plays a role in preparing for impact [12]. In this study, our intervention primarily targeted the stance phase, with no direct interventions during the swing phase. Therefore, our findings suggest that the increased muscle activity was due to impaired movement perception related to deceleration and impact absorption from the late swing phase to initial contact.

Additionally, an improvement in the PSEQ score was observed in this case. The reduction in the stretching sensation around the surgical site and the increase in knee flexion ROM may have contributed to an enhanced sense of self-efficacy regarding pain during stair descent. On the other hand, knee extension limitation persisted. It has been shown that patients with significant flexion contracture before surgery are more likely to develop flexion contracture postoperatively [13]. We hypothesized that reduced extensibility of the hamstrings and gastrocnemius persisted postoperatively, highlighting the need for early interventions to improve knee extension mobility. Additionally, although it was not possible to implement this in this case due to the contralateral knee also exhibiting extension restriction, adjustments in support height should be considered to prevent compensatory extension limitations in the affected knee while standing, caused by leg length discrepancy.

Compared to previous studies that reported changes in knee flexion ROM from before to after surgery, knee flexion ROM in this case had not sufficiently increased by the time of discharge [14]. Increasing evidence suggests that psychosocial factors, such as anxiety, depression, fear of movement, and central sensitization, are significant risk factors for poor prognosis after TKA [15]. The fear-avoidance model postulates that pain-related fear activates escape mechanisms, leading to prolonged avoidance of physical activity. This avoidance exacerbates negative moods, such as depression, and contributes to physical disabilities, including disuse syndrome [16]. It was speculated that the improvement in motor function was not sufficient due to the influence of psychological aspects such as fear of exercise, anxiety, and depression.

While previous studies have investigated the biomechanics of stair descent after TKA using surface EMG, few studies have considered the psychological aspects [17]. In this case, surface EMG was utilized to evaluate both muscle activation and psychological factors affecting stair descent after TKA. When evaluating joint contractions after TKA, previous studies have primarily focused on the vastus medialis and semitendinosus muscles. However, in this case, electrodes were attached to the medial head of the gastrocnemius and the semitendinosus. The hamstrings are typically the primary muscles involved in knee flexion, yet in this case, the medial head of the gastrocnemius exhibited greater muscle activity than the semitendinosus during knee flexion. Due to the limitation of using only two channels of surface EMG in this study, we were unable to assess quadriceps muscle activity. If co-contraction were to be evaluated comprehensively, it would have been necessary to include quadriceps muscle assessment. Notably, previous studies have not reported increased gastrocnemius muscle activity relative to the hamstrings in similar cases. These findings highlight the potential value of integrating surface EMG assessments with psychological evaluations, particularly in patients who exhibit strong fear and anxiety in addition to physical function limitations.

Regarding the effects of rehabilitation on physical function, studies have reported that standard rehabilitation and motor imagery can improve muscle strength and pain [18]. Additionally, electrical stimulation techniques such as neuromuscular electrical stimulation (NMES) and transcutaneous electrical nerve stimulation (TENS) have been shown to reduce postoperative pain following TKA [6]. Therefore, incorporating motor imagery and physical therapy from an early stage may have been beneficial in restoring physical function.

Several psychological interventions have been reported to be effective, including cognitive behavioral therapy, and graded motor imagery training [19]. These approaches are particularly beneficial in cases where psychological problems such as anxiety and depression are present before surgery. In particular, pain neuroscience education has been reported to reduce attentional focus on pain. Among the psychological interventions, pain neuroscience education may have been especially important in this case, as it likely contributed to reducing fear by shifting attention away from pain and facilitating postoperative recovery.

This case report has several limitations. First, it describes a single case, and the evaluation was conducted using a stair height of 20 cm, limiting its statistical generalizability. Second, surface EMG was recorded using only two channels, which restricted the scope of the discussion. Therefore, further studies with larger sample sizes and more comprehensive assessments are necessary. In the future, it will be essential to apply surface EMG to a broader range of muscles and conduct longitudinal psychological evaluations. Additionally, early rehabilitation strategies should integrate both physical and psychological factors to optimize functional recovery.

## Conclusions

This case suggests that fear of stair descent may increase protective-like muscle activity in the semitendinosus and medial head of the gastrocnemius during the swing phase. For TKA patients with pronounced anxiety or fear of movement, it may be necessary to enhance physical function through the implementation of surface EMG assessments, longitudinal psychological evaluations, and targeted psychological interventions based on the results.
